# Effects of Different Protein Feeds on Nutrient Digestion, Energy Metabolism, Methane Emissions, and Rumen Microbiota in Mutton Sheep

**DOI:** 10.3390/ani15233460

**Published:** 2025-11-30

**Authors:** Yiqiang Wang, Zhengxin Zou, Ziwei Wang, Nazir Ahmad Khan, Hangshu Xin, Xiaogang Yan

**Affiliations:** 1 College of Animal Science and Technology, Northeast Agricultural University, Harbin 150030, China; dbnd_wyq@163.com (Y.W.); bobpopdod123@163.com (Z.Z.); wzw17390606858@163.com (Z.W.); 2 Department of Animal Nutrition, The University of Agriculture Peshawar, Peshawar 25130, Pakistan; nazir.khan@aup.edu.pk; 3 Institute of Animal Nutrition and Feed Science, Jilin Academy of Agricultural Sciences, Changchun 136199, China

**Keywords:** energy utilization, methane emissions, protein feed, rumen microorganism, sheep

## Abstract

Making full use of feed nutrients, reducing energy losses and methane emissions, and maximizing production benefits have been ongoing goals of livestock production. Thirty-six healthy Dorper × Thin-tailed Han mutton ewes were divided into six groups using a completely randomized experimental design. A basal diet was provided to the control group, while the experimental group consumed a diet in which 15% was replaced by equal amounts of cottonseed meal (CM), rapeseed meal (RM), distillers dried grains with solubles (DDGS), soybean meal (SM), and fermented soybean meal (FSM). The study demonstrated that SM had the highest protein digestibility, followed by FSM, DDGS, and CM in that order. In terms of net energy, DDGS showed a significantly higher value compared to FSM (*p* < 0.05). Significantly higher abundances of *Lactobacillaceae* and *Bifidobacteriaceae* were observed in the CM group relative to the RM and FSM groups (*p* < 0.05). The methane emissions that we were most concerned about, whether it was daily emissions or metabolic weight basis, showed the same disparity between the groups, with the RM group having the most, followed in order by FSM, DDGS, and SM groups, and the least by the CM group. We found that the RM group had the highest relative abundance of *Methanobrevibacter*, followed by the FSM, SM, DDGS, and CM groups in that order, which is similar to the trend of methane emissions from each group. In summary, the highest apparent protein digestibility is SM, and the highest net energy value among these protein feeds is DDGS. RM will produce the most methane. In addition, the source of protein feed in the diet will affect the structural composition of rumen microorganisms.

## Simple Summary

In livestock production, reducing energy losses and methane emissions, as well as improving the utilization of feed nutritive value, are key issues in maximizing benefits. The study demonstrated that the protein digestibility followed the order: soybean meal (SM) > fermented soybean meal (FSM) > distillers dried grains with solubles (DDGS) > cottonseed meal (CM) > rapeseed meal (RM). The net energy value was highest in DDGS, followed by CM, SM, RM, and FSM. Conversely, methane emissions were most pronounced in RM, followed by FSM, DDGS, SM, and CM. The addition of CM can increase the relative abundance of *Lactobacillaceae* and *Bifidobacteriaceae* and reduce the relative abundance of *Methanobrevibacter*. In the actual application of protein feeds, farmers should apply these protein feeds according to different production purposes and needs so as to realize sustainable development.

## 1. Introduction

The growing global population, coupled with an increasing demand for higher-quality livestock products, has stimulated the expansion of livestock farming, particularly for ruminants such as cattle and sheep [1]. The global beef production is forecast to rise by 0.7% in 2024, totaling 77.2 million tonnes according to the latest Food and Agriculture Organization of the United Nations (FAO) report [2]. Similarly, global mutton production is expected to rise to 17.3 million tonnes, a 0.8% increase. However, this growth in the ruminant industry has further exacerbated significant environmental concerns, particularly regarding methane (CH_4_) emissions. Macrae [3] highlights that ruminants are less than 20% efficient at converting dietary protein into livestock products. Methane is a major greenhouse gas (GHG), accounting for 16% of total GHG emissions [4]. Although its overall emissions are lower than those of carbon dioxide (CO_2_), methane has a greenhouse effect that is 25 times greater per unit volume than CO_2_ [4]. Ruminants are significant non-CO_2_ GHG emitters, with livestock farming estimated to contribute 7.1 billion tonnes of CO_2_-equivalent GHGs, representing 14.5% of anthropogenic emissions-surpassing the transportation sector’s share [5]. Although CH_4_ mainly comes from carbohydrates, the dietary protein content encourages the production of propionic acid, which means more hydrogen is used to create propionic acid instead of CH_4_ [6]. This change in dietary structure and certain anti-nutritional factors can also lead to alterations in the composition of the rumen microbiota community, thereby affecting CH_4_ emissions [7,8]. Energy losses associated with CH_4_ production during rumen fermentation can range from 2% to 12% [9]. Therefore, maintaining an extremely low level of CH_4_ emissions is of vital importance for improving feed utilization efficiency and enhancing productivity.

Protein is a critical nutrient and an essential component for ruminant feed [10,11]. Traditionally, soybean meal (SM) has been the primary source of protein for livestock and poultry due to its favorable nutritional profile and balanced amino acid composition [12,13]. However, rising prices for SM in recent years have significantly increased the costs of livestock feeding, jeopardizing the stability of the entire supply chain, especially in China [14,15]. Mitigating these challenges requires strategic SM reduction and substitution coupled with optimal utilization of local by-product meals, which are critical for cost reduction and enhanced industrial competitiveness [16]. With their rich nutritional profiles, processing by-products including cottonseed meal (CM), rapeseed meal (RM), distillers dried grains with soluble (DDGS) from corn processing, and fermented soybean meal (FSM) are increasingly being incorporated into livestock feed formulations [17,18,19]. Animals require amino acids rather than protein per se for growth and development, and excess protein supply and amino acid imbalance may lead to significant urinary nitrogen excretion and low nitrogen utilization [20]. As competitiveness in modern animal husbandry intensifies, achieving higher economic returns necessitates a scientific approach to livestock management, highlighting the importance of providing well-formulated feed. Ruminants exhibit varying degrees of feed utilization, making it imperative to accurately assess the nutrients and energy content of feed ingredients to optimize protein feed utilization [21,22,23].

The net energy (NE) system is recognized as the most accurate measure of a feed’s effective energy value [24]. Unlike monogastric animals, which can directly assess feed ingredients to determine their nutritional value and energy levels, the unique digestive physiology of ruminants complicates the assessment of many protein feeds [25,26]. Currently, ruminant feeds primarily allow for the direct measurement of conventional nutrients, such as crude protein, while digestible and metabolizable energy cannot be assessed with the same precision. Most digestible and metabolizable energy values reported in feed nutrient tables are derived from calculations or in vitro methods, often lacking correlation with actual in vivo conditions [27,28]. This discrepancy can result in misalignment between formulation applications and the nutritional requirements of animals [29].

To address these challenges, this experiment employed the reference diet substitution method, a widely recognized approach in NE determination for monogastric animal feed ingredients [30,31,32]. The five protein feeds selected for this experiment (SM, FSM, CM, RM, DDGS) were chosen due to their significant differences in protein rumen degradability, anti-nutritional factor content, fiber and fat composition, and price, forming an ideal comparative system. This system aims to reveal the relationship between protein source characteristics and methane production, providing a scientific basis for selecting protein feeds in practical production based on energy utilization, environmental, and economic benefits. Therefore, we hypothesize that these five protein feeds will exert distinct effects on nutritional digestibility, energy metabolism, and CH_4_ emissions in mutton sheep, with rumen microorganisms playing a significant role in these processes.

## 2. Materials and Methods

### 2.1. Animal Ethics Statement

The experiment was conducted at the Institute of Animal Nutrition and Feed Science, Jilin Academy of Agricultural Sciences (Changchun, Jilin, China) between October and December 2022. All procedures were reviewed and approved by the Animal Welfare and Ethics Committee of Northeast Agricultural University (Protocol No. NEAUEC20181007) and were performed in strict compliance with institutional ethical guidelines for animal research.

### 2.2. Protein Feeds

All protein feeds used in this study were obtained from Jilin Jijia Animal Husbandry Technology Co., Ltd., Gongzhuling, China. SM, CM, and RM are all byproducts resulting from the pre-press extraction process used to extract oil from their respective seeds. Additionally, DDGS is a byproduct generated during ethanol production. The FSM was produced through synergistic fermentation with 3% lactic acid bacteria (5 × 10^9^ CFU/mL, *Lactobacillus rhamnosus* CICC 23119) and 0.5% acid protease (60,000 U/g). The nutrient composition of these five protein feeds is detailed in Table 1.

### 2.3. Animals, Diets, and Experimental Design

Thirty-six healthy Dorper × Thin-tailed Han crossbred ewes (55.8 ± 7.34 kg) were randomly assigned to six groups. Animals in the control group were maintained on a basal diet, whereas each experimental group received a modified version with 15% of the diet replaced by one of the test ingredients (CM, RM, DDGS, SM, or FSM). The experimental diets (Table 2) were formulated to NRC [33] standards, meeting maintenance and growth requirements for 55 kg ewes. All animals were housed in individual pens with free access to clean water. The initial 14 days were designated for acclimatization, followed by 7 days in the metabolism cage. The first 4 days in the metabolic cage were for acclimatization, and the last 3 days were reserved for sample collection. Throughout the study, a whole mixed pelleted diet was provided, and each ewe was fed 2 kg (fresh matter basis) daily, divided into two equal portions offered at 7:00 and 17:00.

### 2.4. Measures and Sampling

During the sampling period, the weights of daily offered and leftover feed were recorded. The collected feed and leftovers were analyzed for nutrient content to calculate nutrient intake.

Each metabolic cage featured a mesh-shaped leakage plate at the bottom, equipped with a manure-holding device and perforations to facilitate the separation of feces and urine. For three consecutive days, the feces and urine of each ewe were collected in their entirety each morning before feeding. The total volume of feces excreted was accurately measured and recorded. Fecal samples from each ewe were pooled, homogenized, and split: one portion preserved with 10% (*w*/*v*) of 10% H_2_SO_4_ for nitrogen analysis, and the other oven-dried (55 °C, 48 h), ground (1 mm sieve), and stored at 4 °C for nutrient and energy analysis. Urine was collected in a plastic bucket containing 100 mL of 10% H_2_SO_4_ to prevent uric acid precipitation during storage. Following collection, the total urine volume was measured, homogenized, and gauze-filtered (4 layers). The separated 100 mL filtrate was stored at −20 °C for urine energy analysis.

Simultaneous respiratory calorimetry and digestive metabolism tests were conducted in the same metabolic cage, equipped with an 8-compartment open-circuit respiratory calorimetry system specifically developed by the Jilin Academy of Agricultural Sciences for medium-sized animals. This design allowed direct connection to the calorimetric device, enabling simultaneous measurement of gas exchange from eight ewes while permitting free movement. The system continuously monitored the concentration of oxygen (O_2_), carbon dioxide (CO_2_), and CH_4_ in the respiratory compartment over several days, automatically calculating gas yield and respiratory quotient (RQ) every 20 min. Sensors were obtained from Sensors Europe Gmbh (Erkrath, Germany) for CH_4_ and CO_2_ measurements, and from American Megatrends Inc. (Duluth, USA) for O_2_ monitoring. The ewes were weighed at the study’s start and end, with the average weight recorded as the calorimetric weight for each ewe.

On the final day of the experiment, blood was collected from each ewe via jugular vein puncture into a vacuum tube containing sodium heparin, just before the morning feeding. The blood samples were immediately centrifuged at 3000× *g* for 20 min at 4 °C. The upper plasma layer was collected, dispensed into several 1.5 mL centrifuge tubes, and stored at −20 °C until used for subsequent biochemical analyses. And rumen fluid was collected via gastric tube 3 h after morning feeding and stored at −80 °C.

### 2.5. Sample Analysis

Feed and fecal samples were analyzed according to AOAC [34] methods: dry matter (DM; Method 930.15), crude protein (CP; Method 976.05), ether extract (EE; Method 920.39), and ash content (Method 942.05). Non-protein nitrogen (NPN), neutral detergent insoluble crude protein (NDICP), and acid detergent insoluble crude protein (ADICP) were determined following Licitra et al. [35]. Neutral detergent fiber (NDF) and acid detergent fiber (ADF) were quantified using an Ankom 220 fiber analyzer (Ankom Technology Corp., Macedon, NY, USA) based on Van Soest et al. [36]. Gross energy (GE), fecal energy (FE), and urinary energy (UE) were determined with a fully automated oxygen bomb calorimeter (IKA C3000, IKA-Werke GmbH & Co. KG, Staufen im Breisgau, Germany), in accordance with the manufacturer’s instructions.

Digestibility and energy calculations were performed following the methods described by Feng [37] with the following equations: apparent digestibility of nutrients = (total nutrient intake—nutrients in feces)/total nutrient intake, digestive energy (DE) = gross energy intake (GEI)—FE, and metabolizable energy (ME) = GEI-FE-UE—energy loss from CH_4_ emissions (CH_4_E), where CH_4_ mainly comes from rumen fermentation, the heat of CH_4_ is 39.54 KJ/L, and the total heat production (THP, KJ) = 16.175 × O_2_ consumption (L) + 5.0208 × CO_2_ production (L)—0.958 × UN content (g) × 6.25 − 2.167 × CH_4_ production (L) [38]. Dietary net energy (NE) (MJ/kg) = [(GEI)-FE-UE-CH_4_E-THP (MJ) + maintenance net energy (MJ)]/weight of diet intake (kg) [32]. The maintenance net energy in this experiment was based on our previous value of 271.33 kJ/kg BW^0.75^ for fattening mutton sheep using the regression method of heat production [39].

The apparent digestibility of nutrients in feed ingredients was calculated according to Adeloa [40] using the following equation: apparent digestibility of protein feed nutrients (%) = apparent digestibility of a nutrient in the diet-(100 − x) × apparent digestibility of the nutrient in the base ration]/x. Where: x is the proportion of the ingredient to be measured in the test ration (%). The formula for calculating the feed energy value was informed by the methodology of Barzegar et al. [41]: Effective energy of protein feed (MJ/kg) = (Effective energy of test ration—Effective energy value of base ration × a%)/b%. Where: a is the proportion of the energy-supplying ingredient portion of the base ration in the test ration (%); b is the proportion of the ingredient to be tested in the test ration (%). Energy values include DE, ME, and NE. Additionally, the gas yield of the protein feed was calculated using the aforementioned formula.

A fully automatic biochemical analyzer was used to analyze the total protein (TP), albumin (ALB), globulin (GLB), total cholesterol (TC), triglyceride (TG), high-density lipoprotein (HDL), low-density lipoprotein (LDL), very low-density lipoprotein (vLDL), creatinine (CREA), urea (UREA), glucose (GLU), aspartate aminotransferase (AST), alanine aminotransferase (ALT), and alkaline phosphatase (ALP) in plasma (Mindray BS-420; Shenzhen Mindray Bio-medical Electronics Co. LTD, China).

Rumen fluid ammonia nitrogen (NH_3_-N) was quantified using the phenol-hypochlorite method [42], while volatile fatty acids (VFAs) were analyzed by gas chromatography-mass spectrometry (GC-MS) following Saleem et al. [43].

Following the thawing of rumen fluid samples, DNA extraction was performed using the OMEGA Soil DNA Kit (D5625-01; Omega Bio-Tek, Norcross, GA, USA), adhering to the kit instructions. The primer sequences utilized for PCR amplification of bacteria were: 338F (ACTCCTACGGGGAGGCAGCA) and 806R (GGACTACHVGGGGTWTCTAAT). Given the conservation of the methanogenic archaea mcrA, no specific amplification region exists. The primer sequences chosen for PCR amplification of methanogenic archaea included: mcrA forward primer (GGTGGTGTMGGGATTCACACARTAYGCWACAGC) and mcrA reverse primer (TTCATTGCRTAGTTWGGRTAGTT). Subsequent to PCR amplification, gel recovery purification, and quantification were executed, and online libraries were constructed following the Illumina TruSeq DNA library preparation protocol. Qualified libraries underwent bipartite sequencing on an Illumina NovaSeq machine using the NovaSeq 6000 SP Reagent Kit (500 cycles). Microbiome bioinformatics analysis was conducted using QIIME2 [44], with primer fragments removed using cutadapt [45]. DADA2 [46] was utilized for quality control, denoising, assembly, and de-chimerization. Merged amplicon sequence variants (ASVs) characterized sequences and ASV tables. The QIIME2 classify-sklearn algorithm [47] facilitated the annotation of feature sequences for each ASV using species classification based on the naive Bayes classifier (Greengenes database) [48]. ASV leveling was performed using the QIIME feature-table rarefy function, with leveling depth set to 95% of the minimum sample sequence volume, ensuring that all subsequent analyses were based on the leveled ASV table.

### 2.6. Statistical Analysis

We assessed the normality of the variable distributions with the Shapiro-Wilk test. Data analysis was performed using the PROC MIXED program of SAS (version 9.2; SAS Institute Inc., Cary, NC, USA), according to the formula Y*_ij_* = μ + T*_i_* + S*_j_*+ E*_ij_*, where Y*_ij_* represents the dependent variable, μ is the overall mean, treatments are treated as fixed effects, sheep are treated as random effects, and E*_ij_* is the error term. We used Tukey’s test to correct for multiple comparisons when significant differences were identified. The standard error of the mean represents data variability, and a threshold of *p* < 0.05 was considered statistically significant, and 0.05 < *p* < 0.10 was considered to indicate a trend of change.

## 3. Results

### 3.1. Nutritional Digestion and Rumen Fermentation

The DM and OM intake (Table 3) in the DDGS group was significantly higher compared to the CM group (*p* < 0.05). The additional increase in FSM enabled ewes to consume more CP relative to the CM and DDGS groups (*p* < 0.05). Furthermore, ADF intake was significantly higher in the FSM group than in the CM and RM groups (*p* < 0.05). A non-significant trend (*p* > 0.05) was observed for enhanced CP digestibility in SM and FSM treatments compared to CM, RM, and DDGS dietary regimens. Statistical analysis confirmed that DM digestibility was significantly higher (*p* < 0.05) in the DDGS group than in CM and FSM treatments. SM demonstrated the highest protein digestibility, followed by FSM, DDGS, and CM, respectively. RM exhibited the lowest protein digestibility, which was nearly 12% lower than that of SM.

For ruminal fermentation (Table 4), the pH values in the RM and SM groups were significantly higher than in the CM group (*p* < 0.05). However, the levels of ammoniacal nitrogen, acetate, butyrate, and total VFAs were significantly higher in the CM and FSM groups compared to the other groups (*p* < 0.05). Additionally, propionate and valerate levels were highest in the CM group, significantly exceeding those in the other groups (*p* < 0.05), while the SM group had the highest concentrations of isobutyrate and isovalerate (*p* < 0.05). Regarding the VFA profiles, acetate was the predominant acid in the FSM group, significantly higher than in the CM and SM groups (*p* < 0.05). Conversely, the CM group had the highest percentage of propionate, significantly greater than that in the other groups (*p* < 0.05). Among all groups, the FSM treatment showed the greatest acetate: propionate ratio, with progressively lower values in RM, SM, DDGS, and CM.

### 3.2. Energy Metabolism

In terms of energy metabolism (Table 5), the DDGS group showed the highest daily values of GE, DE, ME, and NE from feeding. Specifically, daily GE and DE were significantly greater in the DDGS group compared to the CM group (*p* < 0.05). Conversely, the RM and CM groups had significantly higher daily UE than the DDGS group (*p* < 0.05). Additionally, daily CH_4_E was significantly lower in the CM group compared to both the RM and FSM groups (*p* < 0.05). As a result of these various energy accumulations and changes, the daily ME in the DDGS group was significantly higher than that of the CM group (*p* < 0.05), while the other protein treatment groups showed little variation. The FSM group exhibited the highest single-day HI, resulting in significantly greater daily NE in the DDGS group compared to the CM, RM, and FSM groups (*p* < 0.05).

For energy conversion, the DDGS group had the highest DE/GE, ME/GE, and NE/GE ratios, although the differences were not significant compared to other protein treatment groups (*p* > 0.05), except for its NE/GE ratio, which was significantly higher than that of the FSM group (*p* < 0.05). By calculating the energy values of each protein feed, the DE and ME of DDGS were significantly higher than those of the other four common protein feeds (*p* < 0.05), with DE in FSM and ME in RM being relatively lower. In terms of NE, the DDGS group was significantly higher than the FSM group (*p* < 0.05), while differences with the other groups were not statistically significant (*p* > 0.05).

### 3.3. Ruminal Bacterial Communities

For alpha diversity (Table S1) and beta diversity (Figure S1), principal coordinates analysis and non-metric multidimensional scaling (NMDS) revealed substantial differences between the CM group and the other experimental groups.

**Figure 1:**
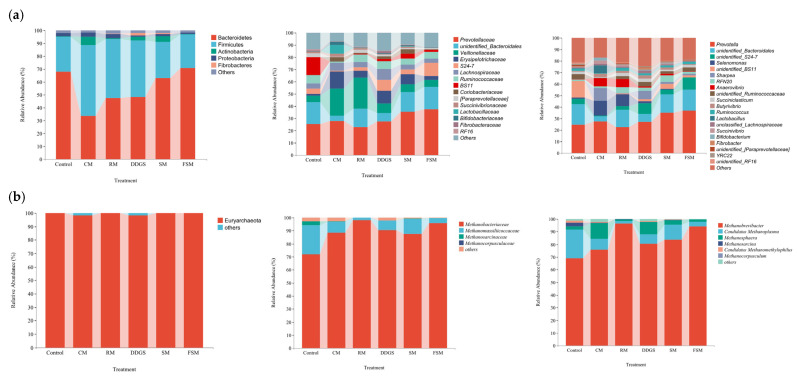
Abundance of ruminal bacterial communities (**a**) and ruminal methanogenic archaea communities (**b**) in mutton sheep fed common protein feeds (Phylum level, family level, genus level).

At the phylum level (Figure 1), Bacteroidetes and Firmicutes were the dominant microbial groups. The abundance of Bacteroidetes (Table 6) in the FSM group was significantly higher than that in the CM group (*p* < 0.05). Conversely, the abundance of Firmicutes in the CM group was significantly higher than in the FSM group (*p* < 0.05). At the family level, the DDGS group exhibited a higher abundance of *Lachnospiraceae* compared to the RM, SM, and FSM groups (*p* < 0.05). However, the CM group had the highest abundance of *Lactobacillaceae* and *Bifidobacteriaceae*, significantly exceeding that of the RM and FSM groups (*p* < 0.05). At the genus level, the abundance of *Succiniclasticum* was significantly lower in the CM group compared to the RM and DDGS groups (*p* < 0.05). Notably, the abundance of *Butyrivibrio* was significantly higher in the CM and DDGS groups than in the FSM groups (*p* < 0.05). Similarly, the CM group had the highest abundance of *Lactobacillus*, which was significantly greater than that in the other groups (*p* < 0.05). In contrast, the abundance of *Bifidobacterium* was significantly higher in the CM group compared to the RM and FSM groups (*p* < 0.05).

### 3.4. Methane Emissions and Methanogenic Archaea Communities

The gas data (Table 7) from the respiratory chamber showed no significant difference in O_2_ consumption among the groups (*p* > 0.05). The RQ of the RM, DDGS, and SM groups was significantly higher than that of the FSM and CM groups (*p* < 0.05), due to significantly higher CO_2_ emission in the DDGS group compared to the CM group (*p* < 0.05). The CH_4_ emissions, of primary concern, were consistently higher in the RM group, followed in descending order by the FSM, DDGS, and SM groups, with the CM group exhibiting the lowest emissions. The differences in gas emissions resulted in significantly higher THP values (BW^0.75^) in the FSM group compared to the CM group (*p* < 0.05). When examining CH_4_ yields among the feeds, emissions ranked in the following order: RM, FSM, DDGS, SM, and CM.

The significant differences in beta diversity were observed among the RM, DDGS, and FSM groups (Figure S1). Overall, methanogens from all groups belonged to the phylum Euryarchaeota (Figure 1). At the family level (Table 7), all protein feed substitution groups showed a significant increase in the relative abundance of *Methanobacteriaceae* compared to the control group (*p* < 0.05), whereas the abundance of *Methanomassiliicoccaceae* decreased. *Methanobrevibacter* abundance ranked RM > FSM > SM > DDGS > CM, matching the methane emission gradient. Additionally, the relative abundances of *Candidatus Methanoplasma* and *Candidatus Methanomethylophilus* decreased in all groups with the replacement of protein feeds, especially in the RM group.

## 4. Discussion

### 4.1. Nutritional Digestion and Rumen Fermentation

To evaluate feed nutritive value and availability, precise nutrient quantification must be complemented by a thorough understanding of metabolic processes and how nutrients are expressed biologically to determine their true availability [49,50]. The protein-enriched feed groups exhibited increased DM, OM, and CP intake, potentially attributable to their greater concentrate proportion. These findings corroborate prior research indicating that higher concentrate levels or energy-dense diets stimulate DM intake in ruminants [51,52]. Furthermore, the total mixed pellet feed utilized in this study promoted the digestion and absorption of feed nutrients while reducing selective eating behavior among sheep, leading to changes in ADF intake attributable to the increase in DM intake. Klusmeyer et al. [53] found that the digestibility of OM, NDF, and ADF in the diets of dairy cows was not significantly affected by changes in the nitrogen source, a finding partially consistent with the results of the present trial. However, Nampoothiri et al. [51] reported that CP digestibility in all medium and high protein level groups was significantly higher than in the low protein level group. This aligns with the findings of the current experiment, which demonstrated a significant improvement in CP digestibility across all treatment groups. Yin et al. [54] similarly found that protein digestibility was significantly reduced when SM was completely replaced by CM, RM, or DDGS in growing lambs. Zhang et al. [55] used FSM as a replacement for SM in fattening lamb diets and found improved digestibility of nutrients, including DM, CP, and NDF, as well as increased nitrogen deposition and utilization.

NH_3_-N represents the terminal product derived from the catabolism of feed proteins, amino acids, urea, and other nitrogenous compounds within the ruminal ecosystem [56]. The concentration of ruminal NH_3_-N exhibits significant correlations with multiple factors, including dietary protein level, protein degradation characteristics, microbial protein synthesis efficiency, and chyme evacuation rate [57]. The NH_3_-N generated through ruminal fermentation undergoes partial utilization for microbial protein synthesis, while the remaining protein exists in a free state. The digestive epithelium absorbs this free NH_3_-N, which then enters portal circulation and is transported to the liver for urea cycle metabolism. [58]. This explains the increase in plasma urea content in addition to protein levels due to the addition of protein feeds in treatment groups (Table S2). Our results indicate NH_3_-N levels in the CM group was significantly increased, potentially due to two primary factors: (1) the presence of cotton phenols that may enhance the degradation rate of nitrogenous compounds beyond the microbial capacity to NH_3_-N utilization [59]; and (2) the elevated NPN content in CM formulations, which undergoes rapid ruminal decomposition, thereby contributing to NH_3_-N accumulation.

Ruminal pH is regulated by multiple interrelated factors, including dietary composition, temporal patterns following feeding, absorption of VFA through the rumen epithelium, acid efflux mechanisms, and salivary buffer secretion rates [60]. The physiological range for rumen pH typically spans from 5.5 to 7.5 [60,61]. In the current trial, all treatments maintain rumen pH within this normal range, indicating the absence of adverse effects on ruminal pH. The rapid fermentation of fermentable carbohydrates post-ingestion generates substantial quantities of lactic acid and VFAs, which may surpass the combined absorption capacity of the rumen epithelium and the buffering capacity of salivary secretions, ultimately leading to acid accumulation and pH depression [62]. The production dynamics and molar proportions of VFAs significantly affect nutrient absorption efficiency and overall ruminant productivity [19]. Our experiment results demonstrated distinct rumen fermentation patterns among different protein feed components. A similar trend was observed for propionate content across all five protein sources, contrasting with the findings of Yin et al. [54] that indicated no change. These observed variations may be attributed to interspecies differences and the inherent nutritional variability among feed ingredients. Supporting this observation, Research by Pang et al. [63] revealed that higher concentrate levels stimulate rumen microbial fermentation, leading to elevated yields of acetate, propionate, and butyrate. Among all treatments, CM produced the highest levels of acetate, propionate, butyrate, valerate, and TVFA, demonstrating optimal fermentation performance. This enhanced fermentation efficiency may be associated with CM, which appears to modulate rumen bacterial community structure more effectively than other protein sources [64]. Our results revealed significant alterations in rumen microbiota following CM substitution, characterized by increased relative abundance of *Butyrivibrio*, *Lactobacillus,* and *Bifidobacterium*. These microbial shifts potentially explain the observed increases in VFA fractions and total VFA contents, coupled with the pH reduction.

### 4.2. Energy Metabolism

Energy serves as the fundamental driver of animal life processes and production activities, representing one of the most essential nutritional components [65,66]. The dietary constituents, including proteins, carbohydrates, and lipids, primarily function as energy sources for animals. Through a series of physiological and biochemical transformations, these macronutrients release energy in the form of ATP, thereby meeting the organism’s metabolic demands [67]. In the current investigation, the DDGS and FSM groups exhibited significantly higher total energy intake, attributable to the synergistic effects of enhanced feed consumption and elevated dietary energy density in these treatment groups. According to the principle of energy conservation, the majority of this energy supports vital biological processes such as growth, fat deposition, gestation, and lactation, while a portion is inevitably lost through fecal excretion, urinary output, gaseous emission, and heat dissipation [68]. The FE represented the predominant pathway of energy loss, accounting for approximately one-third of total energy expenditure, a pattern consistently observed across all groups. The DDGS group exhibited the most pronounced DE and DE digestibility. While DE represents the actual energy assimilated by monogastric animals and serves as an indicator of feed digestibility [69], its application in ruminant nutrition requires careful consideration. The unique digestive physiology of ruminants, characterized by microbial fermentation in the reticulo-rumen, results in significant energy losses through fermentation heat and CH_4_ production. These inherent energy losses render the DE system less suitable for evaluating energy utilization in mutton sheep production systems [69].

Furthermore, our observations revealed elevated urinary energy values and corresponding percentages in RM and SM groups, which may be attributed to the dietary CP and metabolizable protein concentrations exceeding the animal’s physiological requirements. The resultant nitrogen surplus undergoes hepatic conversion to urea, uric acid, and creatinine, which are subsequently excreted through urinary pathways, thereby increasing urinary energy output [70,71,72]. Regarding CH_4_ emissions, the energy values were directly proportional to the animals’ CH_4_ production. Notably, the RM and FSM groups exhibited significantly higher daily CH_4_ energy values compared to the CM group. However, when expressed as a percentage of GE, CH_4_ energy ranged between 3.68% and 5.43%, consistent with values reported in comparable studies [73,74].

The total energy digestibility and total energy metabolism ratios ranged from 64.9% to 67.6% and 57.4% to 61.4%, respectively. These values were comparatively higher than those reported by Cui et al. [73], who observed ranges of 56% to 65% for total energy digestibility and 48% to 59% for total energy metabolism. The higher dietary concentrate proportion likely enhanced energy efficiency, thereby improving overall energy utilization in our study. Among the experimental groups, the DDGS group exhibited superior total energy digestibility and metabolism compared to other protein treatment groups. This enhanced performance was partly associated with DDGS’s distinctive nutritional composition, particularly its elevated fat content and high conversion efficiency. The metabolic advantage of dietary fats is well-documented, as they contain more than twice the energy density per gram compared to carbohydrates and proteins [75]. Furthermore, the metabolic pathway of fat utilization demonstrates higher efficiency, with nearly all consumed fat being stored, in contrast to carbohydrates and proteins, which experience significant energy loss during absorption, processing, and storage. This fundamental difference in energy metabolism explains the superior energy utilization observed in the DDGS group [75,76].

In contrast to both ME and DE systems, the NE system provides a more comprehensive assessment by considering not only the energy losses occurring during digestion and metabolic processes of feed within the organism, but also the energy expenditure required to facilitate these physiological processes [77]. Consequently, the implementation of the NE system represents an essential approach for precision mutton sheep farming and the optimization of economic efficiency in this agricultural sector [78]. Our results revealed distinct variations in NE production among different groups. The DDGS group showed superior NE advantages, while the FSM group demonstrated lower NE production, which is due to its higher heat gain, where a large amount of energy is lost with the higher total heat production due to respiration, allowing a small amount of energy to be accumulated in the body [79].

### 4.3. Ruminal Bacterial Communities

The diversity and relative abundance of rumen bacteria were affected by diet composition, animal species, and developmental stage [80]. Among all protein replacement regimens, the FSM supplementation consistently showed the highest *Prevotellaceae* proliferation. *Prevotellaceae* mainly metabolize hemicellulose, pectin, and protein in the rumen, and mainly produce acetic acid and formic acid [81]. This increase in relative abundance not only improves proteolytic efficiency but also improves nitrogen utilisation and reduces nitrogen pollution in the environment [82]. Nitrogen that is not utilised during livestock farming can cause eutrophication of water bodies, degradation of soil, acidification, and atmospheric pollution. Nitrous oxide (N_2_O), which nitrogen is converted to through denitrification, has a global warming potential (GWP) that is 265 times that of CO_2_, much higher than that of methane [83]. This study independently validated Wang et al.’s [84] findings, demonstrating that FSM substitution consistently modulates rumen microbiota toward *Prevotella*-dominated communities with enhanced Succiniclasticum populations. Xie et al. [85] similarly reported elevated Bacteroidetes populations and *Prevotella* enrichment in the hindgut of weaned piglets receiving fermented soybean meal. *Lachnospiraceae* and *Ruminococcaceae* are thought to be highly specialized in degrading feed to VFA in the rumen [86]. *Lachnospiraceae* play a leading role in rumen biohydrogenation, and in particular, *Butyrivibrio* have been identified as the most important biohydrogenating bacteria [87]. *Butyrivibrio* represents a unique rumen bacterial genus with demonstrated biohydrogenation capacity, serving as a key regulator of ruminal lipid metabolism [88,89]. The elevated EE and unsaturated fatty acid content in DDGS [90] likely promoted the enrichment of *Lachnospiraceae* and *Butyrivibrio*, thereby enhancing fatty acid digestibility through their biohydrogenation activities. *Butyrivibrio* is one of the important fibre-decomposing bacteria in the rumen, and also the most important butyrate-producing bacteria in the rumen [91]. This may explain the increase in butyrate concentration in rumen fluid with the addition of CM and DDGS. The elevated abundance of these bacterial taxa enhanced ruminal VFA production and improved energy utilization efficiency in mutton sheep, aligning with the superior energy utilization observed for DDGS and CM. *Ruminococcaceae* participate in the degradation of fibers in the rumen, and the increase of its relative abundance can significantly improve the digestibility of dietary NDF [92]. While *Ruminococcaceae* abundance was elevated in RM, DDGS, SM, and FSM groups compared to CM, no corresponding improvement in NDF digestibility was detected. However, it is worth mentioning that methane emissions are positively correlated with fibre digestibility [93].

Both *Lactobacillaceae* and *Bifidobacteriaceae* belong to the probiotic family [94]. Members of the *Lactobacillaceae* family have been proposed to play a key role in host metabolic homeostasis by protecting intestinal integrity from pathogen disruption and can reduce inflammation [95]. *Lactobacillus*, which was significantly increased in the CM group animals, has been shown to produce essential short-chain fatty acids, including lactic acid and acetate, which promote MCP production in the rumen [96,97]. Raffinose is an oligosaccharide composed of galactose, glucose, and fructose and is found mainly in dietary protein sources such as CM, SM, and corn [98,99]. The raffinose content of CM was higher at 6.52% and SM contained 1.0–2.2% [99,100]. FSM reduces soy oligosaccharides, stachyose, and raffinose in SM due to the fermentation process with bacteria [101]. Smiricky et al. [102] showed a significant increase in fecal *Bifidobacterium* and *Lactobacillus* with dietary addition of soy solubles (sucrose, fructose, and raffinose). Pacifici et al. [103] injected raffinose into the amniotic cavities of chicken embryos and found that raffinose significantly increased the number of *Bifidobacterium* and *Lactobacillus* in the intestinal tract. Lactic acid undergoes secondary ruminal fermentation via lactate-utilizing bacteria, generating propionate while consuming electrons that would otherwise contribute to methanogenesis [104]. In addition, lactate conversion to propionate concomitantly depletes ruminal hydrogen, directly curtailing methane production through the hydrogenotrophic pathway [105]. And redirecting rumen fermentation toward propionate production concomitantly suppresses hydrogenogenic bacterial activity [106].

### 4.4. Methane Emissions

Methane production in ruminants is primarily influenced by several key factors, including breed and genetic characteristics, physiological stage, and the nutritional composition and source of dietary components [107]. The metabolic process involves the conversion of CO_2_, H_2,_ and acetic acid, which are byproducts of microbial fermentation by rumen bacteria, protozoa, and fungi, into methane through the activity of methanogenic bacteria [108]. The current experiment demonstrated that most protein feed supplements elevated CH_4_ emissions in mutton sheep. Abbasi et al. [109] reported a 50% increase in methane production in Saanen goats when dietary protein content was elevated by 2%. Furthermore, Wolin et al. [110] concluded that the CH_4_ energy loss varies significantly depending on the acetate: propionate ratio, with no energy loss occurring at a ratio of 0.5 and up to 33% loss when carbohydrate fermentation produces only acetate without propionate. This theoretical framework is supported by our data, wherein CM exhibited the lowest CH_4_ production among the tested protein feeds, coinciding with its lowest acetate: propionate ratio.

Methane emissions in ruminants predominantly originate from rumen fermentation processes, contributing to over 80% of total CH_4_ production. Notably, methanogenic archaea are responsible for more than 90% of this CH_4_ output, underscoring the critical need to investigate the composition and diversity of rumen methanogenic communities [111]. In this study, *Methanobacteriaceae* and *Methanobrevibacter* emerged as the dominant archaeal taxa in the rumen fluid across all five protein feed treatment groups, a finding consistent with previous research [112]. Furthermore, our analysis revealed a strong correlation between CH_4_ emissions and the relative abundance patterns of *Methanobacteriaceae* and *Methanobrevibacter* in the rumen fluid. This observation aligns with the work of Gonzalez-Recio et al. [113], who investigated the influence of host genotype on rumen microbial composition in Holstein and Swiss Brown cattle. Their study demonstrated a significant association between *Methanobrevibacter*, *Methanococcus*, and CH_4_ emissions, while also highlighting the impact of animal species on *Methanobrevibacter* populations. Additionally, research has identified inhibitory effects of lactic acid-producing bacteria, such as *Lactobacillus*, on methanogenic activity, leading to reduced CH_4_ emissions [114]. This mechanism likely contributed to the lower CH_4_ emissions observed in the CM treatment group in our experiment.

World farm-gate GHG emissions in 2022 increased by 15% from 2000 to 7.8 billion tonnes CO_2_eq, of which 54% originated from livestock and poultry, particularly enteric fermentation emissions amounting to 2.9 Gt CO_2_eq, accounting for 37% of world farm-gate GHG emissions [2]. Meanwhile, the commodities with the highest concentrations of CO_2_eq emissions are beef and mutton. The FAO forecasts that by 2033, global poultry, pork, beef, and mutton consumption are expected to grow by 16%, 8%, 11% and 16% respectively, and world meat production is projected to grow by 12% (41 million tonnes) to 391 million tonnes, with GHG emissions from livestock increasing by 6% [115]. Therefore, our findings provide valuable insights into CH_4_ emission patterns associated with livestock production. Specifically, the study revealed significant variations in CH_4_ emissions observed in diets containing RM, followed by FSM, DDGS, and SM. Notably, CM demonstrated the lowest CH_4_ emissions among the tested protein sources.

## 5. Conclusions

It can be seen that different protein feeds vary greatly in nutrient digestion, energy metabolism, and methane emission in mutton sheep, and will alter rumen fermentation patterns and rumen microbiota structure to varying degrees. In summary, it can be concluded from our results that: (1) The highest apparent digestibility of protein was observed with SM as protein source, followed by FSM, DDGS, and CM in that order, and RM was the lowest. (2) DDGS as a protein source resulted in the highest net energy of the diets, followed by CM, SM, and RM, while FSM was the lowest. (3) Diets with RM as protein source had the highest methane emissions, followed by FSM, DDGS, and SM, while CM had the lowest methane emissions. (4) The addition of CM can increase the relative abundance of *Lactobacillaceae* and *Bifidobacteriaceae* and reduce the relative abundance of *Methanobrevibacter*. It is equally important to note that while substituting SM with CM, RM, DDGS, or FSM holds potential in terms of economics or nutritional composition, this potential is constrained by the combined factors of feed prices, nutritional content, environmental policies, and specific farm conditions. However, translating this potential into commercial practice will require long-term, large-scale in vivo feeding trials in the near future to evaluate the synergistic effects with other feeds on production performance, animal health, and overall economic benefits. Concurrently, developing rapid, low-cost feed quality testing technologies is crucial for stabilizing the application of these highly variable by-products.

## Figures and Tables

**Table 1:** Nutrient levels of common protein feeds.

Item	CM	RM	DDGS	SM	FSM
DM, %	89.0	89.9	89.2	87.4	90.1
CP, % DM	49.0	40.8	26.6	43.3	49.7
Ash, % DM	8.73	9.83	5.84	6.37	6.55
NDF, % DM	16.2	20.6	25.5	9.48	17.9
ADF, % DM	11.8	13.4	9.10	8.20	10.9
NPN, % DM	1.27	1.63	1.24	1.32	0.74
NDICP, % DM	4.62	5.90	4.57	1.59	6.56
ADICP, % DM	0.83	2.84	2.51	1.63	0.92
EE, % DM	1.05	0.56	10.0	0.98	0.56
GE, MJ/kg	17.6	17.3	19.6	17.2	17.7

CM = Cottonseed meal; RM = Rapeseed meal; DDGS = Distillers dried grains with soluble; SM = Soybean meal; FSM = Fermented soybean meal. The same as below.

**Table 2:** Feed formulation and its nutrient levels of different diets.

Item	Control	CM	RM	DDGS	SM	FSM
Ingredients, %						
Corn stalk	28.48	23.95	23.95	23.95	23.95	23.95
Corn	26.14	21.98	21.98	21.98	21.98	21.98
Wheat bran	15.0	12.61	12.61	12.61	12.61	12.61
Soybean meal	11.6	9.75	9.75	9.75	24.75	9.75
Cottonseed meal	0	15.0	0	0	0	0
Rapeseed meal	0	0	15.0	0	0	0
Distillers’ dried grains with soluble	0	0	0	15.0	0	0
Fermented soybean meal	0	0	0	0	0	15.0
Alfalfa meal	10.0	8.41	8.41	8.41	8.41	8.41
Molasses	3.00	2.52	2.52	2.52	2.52	2.52
Premix ^1^	2.50	2.50	2.50	2.50	2.50	2.50
Limestone	1.41	1.41	1.41	1.41	1.41	1.41
Calcium hydrogen phosphate	1.17	1.17	1.17	1.17	1.17	1.17
Salt	0.70	0.70	0.70	0.70	0.70	0.70
Total	100	100	100	100	100	100
Nutrient levels, % DM ^2^						
GE, MJ/kg	14.8	15.6	15.4	15.5	15.3	15.4
DM	87.1	90.4	89.3	88.6	89.1	88.5
CP	12.9	18.3	17.5	15.0	17.7	18.9
NDF	29.0	30.2	29.6	28.5	27.6	28.2
ADF	16.7	17.2	15.3	16.0	17.8	18.0
EE	0.59	0.66	0.78	1.16	0.49	0.70
NPN	11.5	16.2	16.1	13.2	15.7	15.8

^1^ Premix provided the following per kilogram of diet: vitamin A 14,000 IU, vitamin E 87.5 mg, vitamin D3 700 IU, Fe 175 mg, Cu 17.5 mg, Mn 105 mg, Se 0.3 mg, Co 0.7 mg. ^2^ Nutrient levels were calculated values.

**Table 3:** Intake and apparent digestibility of nutrients in mutton sheep fed common protein feeds.

Item	Treatment						SEM	*p*-Value
	Control	CM	RM	DDGS	SM	FSM		
Intake								
DM, g/d	1349 ^b^	1351 ^b^	1444 ^ab^	1522 ^a^	1436 ^ab^	1474 ^ab^	38.2	0.02
OM, g/d	1166 ^b^	1172 ^b^	1247 ^ab^	1323 ^a^	1253 ^ab^	1280 ^ab^	33.2	0.02
CP, g/d	200 ^c^	273 ^b^	283 ^ab^	258 ^b^	286 ^ab^	314 ^a^	7.83	<0.01
NDF, g/d	449	452	479	489	445	469	12.2	0.08
ADF, g/d	259 ^bc^	257 ^bc^	248 ^c^	275 ^abc^	287 ^ab^	300 ^a^	7.6	<0.01
Digestibility, %								
DM	61.7	61.6	63.3	64.2	63.7	61.9	0.87	0.17
OM	64.8	65.5	67.1	68.3	68.2	66.1	0.91	0.06
CP	72.1 ^b^	73.8 ^ab^	73.7 ^ab^	74.4 ^ab^	75.5 ^a^	75.3 ^a^	0.70	0.02
NDF	40.8	43.2	43.2	42.2	43.3	43.7	2.04	0.93
ADF	31.2	30.8	29.5	32.0	34.2	34.0	3.20	0.87
Feed digestibility, %								
DM	-	60.8 ^b^	72.1 ^ab^	78.4 ^a^	75.1 ^ab^	62.9 ^b^	3.08	0.01
OM	-	72.9	79.7	87.8	87.1	73.5	3.72	0.06
CP	-	83.2 ^ab^	82.7 ^b^	87.3 ^ab^	95.0 ^a^	93.3 ^ab^	2.56	0.02
NDF	-	56.6	56.8	49.9	57.5	59.8	6.99	0.88
ADF	-	28.5 ^bc^	20.1 ^c^	36.9 ^abc^	51.3 ^a^	49.8 ^ab^	6.58	0.04

^a–c^ Values in the same line with different capital letter superscripts mean samples have significant differences. SEM, standard error of the mean.

**Table 4:** Ruminal fermentation in mutton sheep fed common protein feeds.

Item	Treatment						SEM	*p*-Value
	Control	CM	RM	DDGS	SM	FSM		
pH	6.88 ^ab^	6.74 ^b^	7.62 ^a^	6.93 ^ab^	7.54 ^a^	7.26 ^ab^	0.163	0.01
NH_3_-N, mg/100 mL	18.4 ^b^	31.3 ^a^	13.3 ^b^	15.6 ^b^	19.1 ^b^	25.9 ^a^	1.51	<0.01
Acetate, mg/L	543 ^b^	678 ^a^	388 ^c^	502 ^b^	435 ^c^	680 ^a^	12.3	<0.01
Propionate, mg/L	257 ^cd^	649 ^a^	240 ^d^	328 ^b^	284 ^bcd^	324 ^bc^	14.5	<0.01
Butyrate, mg/L	269 ^b^	461 ^a^	229 ^b^	285 ^b^	280 ^b^	428 ^a^	20.2	<0.01
Isobutyrate, mg/L	29.1 ^b^	32.2 ^ab^	46.7 ^ab^	37.2 ^ab^	60.8 ^a^	53.6 ^ab^	5.98	0.02
Valerate, mg/L	25.0 ^b^	162 ^a^	31.8 ^b^	37.3 ^b^	43.3 ^b^	42.4 ^b^	6.20	<0.01
Isovalerate, mg/L	43.0 ^c^	31.5 ^c^	70.1 ^b^	56.1 ^bc^	98.4 ^a^	77.8 ^ab^	5.23	<0.01
TVFA, mg/L	1167 ^c^	2012 ^a^	1005 ^d^	1246 ^c^	1202 ^c^	1605 ^b^	30.6	<0.01
Proportion of VFA, %								
Acetate	46.6 ^a^	34.9 ^c^	38.7 ^bc^	40.3 ^bc^	36.2 ^c^	42.4 ^ab^	1.21	<0.01
Propionate	22.0 ^bc^	33.4 ^a^	23.9 ^bc^	26.4 ^b^	23.6 ^bc^	20.2 ^c^	1.11	<0.01
Butyrate	23.1	22.9	22.6	22.9	23.3	26.6	1.37	0.37
Isobutyrate	2.49 ^c^	1.70 ^c^	4.63 ^ab^	2.99 ^bc^	5.05 ^a^	3.34 ^bc^	0.342	<0.01
Valerate	2.14 ^b^	8.36 ^a^	3.19 ^b^	2.97 ^b^	3.60 ^b^	2.65 ^b^	0.499	<0.01
Isovalerate	3.67 ^b^	1.64 ^c^	6.97 ^a^	4.51 ^b^	8.18 ^a^	4.84 ^b^	0.364	<0.01
Acetate: propionate	2.13 ^a^	1.05 ^d^	1.65 ^bc^	1.53 ^c^	1.53 ^c^	2.10 ^ab^	0.097	<0.01

^a–d^ Values in the same line with different capital letter superscripts mean samples have significant differences. SEM, standard error of the mean.

**Table 5:** Energy metabolism of mutton sheep fed common protein feeds.

Item	Treatment						SEM	*p*-Value
	Control	CM	RM	DDGS	SM	FSM		
Metabolic weight, kg BW^0.75^	19.8	21.9	20.6	20.9	19.9	19.2	0.83	0.29
Energy values								
GE, MJ/d	23.0 ^b^	23.3 ^b^	25.0 ^ab^	26.7 ^a^	24.7 ^ab^	25.7 ^ab^	0.66	<0.01
GE, MJ/d/kg BW^0.75^	1.17 ^ab^	1.07 ^b^	1.22 ^ab^	1.28 ^a^	1.26 ^ab^	1.34 ^a^	0.044	<0.01
FE, MJ/d	8.56	8.03	8.44	8.65	8.24	9.03	0.309	0.32
FE, MJ/d/kg BW^0.75^	0.43 ^ab^	0.37 ^b^	0.41 ^ab^	0.42 ^ab^	0.42 ^ab^	0.47 ^a^	0.017	0.01
DE, MJ/d	14.4 ^c^	15.3 ^bc^	16.5 ^ab^	18.0 ^a^	16.5 ^ab^	16.7 ^ab^	0.45	<0.01
DE, MJ/d/kg BW^0.75^	0.73 ^ab^	0.70 ^b^	0.81 ^ab^	0.87 ^a^	0.84 ^a^	0.87 ^a^	0.031	<0.01
UE, MJ/d	0.52 ^ab^	0.56 ^ab^	0.76 ^a^	0.39 ^b^	0.69 ^a^	0.59 ^ab^	0.070	0.01
UE, MJ/d/kg BW^0.75^	0.03 ^ab^	0.03 ^ab^	0.04 ^a^	0.02 ^b^	0.04 ^a^	0.03 ^ab^	0.004	0.01
CH_4_E, MJ/d	0.93 ^b^	0.90 ^b^	1.35 ^a^	1.26 ^ab^	1.17 ^ab^	1.27 ^a^	0.085	<0.01
CH_4_E, MJ/d/kg BW^0.75^	0.05 ^ab^	0.04 ^b^	0.07 ^a^	0.06 ^ab^	0.06 ^ab^	0.07 ^a^	0.005	0.01
ME, MJ/d	13.0 ^b^	13.8 ^b^	14.4 ^ab^	16.4 ^a^	14.6 ^ab^	14.8 ^ab^	0.45	<0.01
ME, MJ/d/kg BW^0.75^	0.66 ^b^	0.63 ^c^	0.71 ^abc^	0.79 ^a^	0.74 ^abc^	0.77 ^ab^	0.028	<0.01
HI, MJ/d	4.12 ^b^	4.48 ^b^	5.09 ^ab^	5.08 ^ab^	5.13 ^ab^	6.00 ^a^	0.395	0.01
HI, MJ/d/kg BW^0.75^	0.21 ^b^	0.21 ^b^	0.25 ^ab^	0.25 ^ab^	0.26 ^ab^	0.31 ^a^	0.021	<0.01
NE, MJ/d	8.86 ^b^	9.34 ^b^	9.35 ^b^	11.3 ^a^	9.48 ^ab^	8.85 ^b^	0.503	0.01
NE, MJ/d/kg BW^0.75^	0.45 ^ab^	0.43 ^b^	0.46 ^ab^	0.54 ^a^	0.48 ^ab^	0.46 ^ab^	0.026	0.03
Energy conversion, %								
DE/GE	62.8 ^b^	65.6 ^ab^	66.2 ^ab^	67.6 ^a^	66.6 ^a^	64.9 ^ab^	0.80	0.01
ME/GE	56.5 ^b^	59.3 ^ab^	57.8 ^ab^	61.4 ^a^	59.0 ^ab^	57.6 ^ab^	0.99	0.03
NE/GE	38.6 ^ab^	40.3 ^ab^	37.4 ^ab^	42.4 ^a^	38.2 ^ab^	34.3 ^b^	1.88	0.03
CH_4_E/GE	4.05 ^ab^	3.86 ^b^	5.43 ^a^	4.73 ^ab^	4.79 ^ab^	4.97 ^ab^	0.355	0.04
Feed energy metabolism, MJ/kg DMI								
DE	-	14.8 ^b^	15.7 ^b^	18.3 ^a^	15.8 ^b^	14.7 ^b^	0.58	<0.01
ME	-	13.7 ^b^	12.0 ^b^	17.2 ^a^	13.2 ^b^	12.4 ^b^	0.79	<0.01
NE	-	9.10 ^ab^	5.91 ^ab^	12.2 ^a^	6.62 ^ab^	2.58 ^b^	1.911	0.01

^a–c^ Values in the same line with different capital letter superscripts mean samples have significant differences. SEM, standard error of the mean.

**Table 6:** Abundance of ruminal bacterial communities in mutton sheep fed common protein feeds (%).

Item	Treatment						SEM	*p*-Value
	Control	CM	RM	DDGS	SM	FSM		
Phylum level								
Bacteroidetes	68.0 ^a^	33.5 ^b^	47.4 ^ab^	48.2 ^ab^	62.7 ^ab^	70.9 ^a^	6.06	0.01
Firmicutes	27.0 ^b^	55.1 ^a^	45.7 ^ab^	44.0 ^ab^	28.5 ^ab^	25.8 ^b^	5.50	0.02
Family level								
*Lachnospiraceae*	4.20 ^b^	6.71 ^ab^	4.25 ^b^	9.12 ^a^	3.79 ^b^	3.48 ^b^	0.996	0.001
*Ruminococcaceae*	6.82 ^a^	0.79 ^b^	5.84 ^ab^	6.10 ^ab^	4.94 ^ab^	5.36 ^ab^	1.151	0.04
*Lactobacillaceae*	0.07 ^b^	7.00 ^a^	0.03 ^b^	0.04 ^b^	0.06 ^b^	0.11 ^b^	1.212	0.01
*Bifidobacteriaceae*	0.23 ^b^	2.66 ^a^	0.10 ^b^	0.62 ^ab^	0.85 ^ab^	0.13 ^b^	0.472	0.02
Genus level								
*Succiniclasticum*	2.08 ^ab^	0.98 ^b^	2.97 ^a^	2.93 ^a^	1.45 ^ab^	2.26 ^ab^	0.425	0.02
*Butyrivibrio*	0.98 ^b^	2.84 ^a^	1.61 ^ab^	2.65 ^a^	1.91 ^ab^	0.82 ^b^	0.530	0.046
*Lactobacillus*	0.07 ^b^	6.90 ^a^	0.03 ^b^	0.04 ^b^	0.06 ^b^	0.11 ^b^	1.201	0.01
*Bifidobacterium*	0.01 ^b^	2.60 ^a^	0.09 ^b^	0.60 ^ab^	0.83 ^ab^	0.09 ^b^	0.468	0.02

^a,b^ Values in the same line with different capital letter superscripts mean samples have significant differences. SEM, standard error of the mean.

**Table 7:** Gas metabolism, methane emission, and abundance of ruminal methanogenic archaea communities in mutton sheep fed common protein feeds.

Item	Treatment						SEM	*p*-Value
	Control	CM	RM	DDGS	SM	FSM		
O_2_ consumption, L/d	456	512	514	513	505	543	20.8	0.11
CO_2_ production, L/d	441 ^c^	454 ^bc^	505 ^ab^	511 ^a^	501 ^ab^	504 ^ab^	19.2	0.04
RQ	0.99 ^a^	0.89 ^c^	0.99 ^a^	1.00 ^a^	1.00 ^a^	0.93 ^b^	0.018	<0.01
CH_4_ emission								
CH_4_, L/d	23.6 ^b^	22.8 ^b^	34.1 ^a^	31.9 ^ab^	29.6 ^ab^	32.2 ^a^	2.16	<0.01
CH_4_, L/kg DMI	17.4 ^ab^	16.8 ^b^	23.7 ^a^	20.9 ^ab^	20.8 ^ab^	21.9 ^ab^	1.55	0.03
CH_4_, L/kg BW^0.75^	1.19 ^ab^	1.04 ^b^	1.69 ^a^	1.53 ^ab^	1.55 ^ab^	1.68 ^a^	0.131	0.01
THP, MJ/d	9.49	10.4	10.7	10.7	10.5	11.2	0.48	0.15
THP, MJ/kg BW^0.75^	0.48 ^b^	0.48 ^b^	0.52 ^ab^	0.52 ^ab^	0.53 ^ab^	0.58 ^a^	0.021	<0.01
Feed gas metabolism								
CH_4_ emission, L/kg	-	13.3 ^d^	59.5 ^a^	40.8 ^bc^	40.1 ^c^	47.1 ^b^	1.58	<0.01
Abundance of ruminal methanogenic archaea communities (%)								
family level								
*Methanobacteriaceae*	71.7 ^b^	88.4 ^a^	98.0 ^a^	90.4 ^a^	87.4 ^a^	95.9 ^a^	2.86	0.001
*Methanomassiliicoccaceae*	22.6 ^a^	8.46 ^b^	1.87 ^b^	7.41 ^b^	11.7 ^ab^	3.68 ^b^	2.661	0.003
genus level								
*Methanobrevibacter*	68.9 ^b^	75.8 ^ab^	96.5 ^a^	80.5 ^ab^	83.8 ^ab^	94.2 ^a^	5.09	0.02
*Candidatus Methanoplasma*	22.6 ^a^	8.46 ^b^	1.87 ^b^	7.41 ^b^	11.7 ^ab^	3.68 ^b^	2.66	<0.01
*Candidatus* *Methanomethylophilus*	1.42 ^a^	0.64 ^ab^	0.04 ^b^	0.46 ^ab^	0.47 ^ab^	0.22 ^ab^	0.29	0.04

^a–d^ Values in the same line with different capital letter superscripts mean samples have significant differences. SEM, standard error of the mean.

## Data Availability

The datasets presented in this study can be found in online repositories (NCBI, PRJNA1370297).

## Supplementary Materials

The following supporting information can be downloaded at: https://www.mdpi.com/article/10.3390/ani15233460/s1. Table S1: Alpha diversity of ruminal bacterial communities and methanogenic archaea communities in mutton sheep fed common protein feeds; Table S2: Plasma biochemical indices in mutton sheep fed common protein feeds; Figure S1: Beta diversity of ruminal bacterial communities (a) and ruminal methanogen communities (b) in mutton sheep fed common protein feeds.

## Abbreviations

The following abbreviations are used in this manuscript:

ADF	Acid detergent fiber
ADICP	Acid detergent insoluble crude protein
CH_4_	Methane
CM	Cottonseed meal
CO_2_	Carbon dioxide
CP	Crude protein
DDGS	Distillers dried grains with solubles.
DE	Digestive energy
DM	Dry matter
EE	Ether extract
FE	Fecal energy
FSM	Fermented soybean meal
GE	Gross energy
GHG	Greenhouse gas
ME	Metabolizable energy
NDF	Neutral detergent fiber
NDICP	Neutral detergent insoluble crude protein
NE	Net energy
NH_3_-N	Ammonia nitrogen
NPN	Non-protein nitrogen
O_2_	Oxygen
RM	Rapeseed meal
RQ	Respiratory quotient
SM	Soybean meal
THP	Total heat production
UE	Urinary energy
VFA	Volatile fatty acid
