# Supplementary material for: Effects of Different Protein Feeds on Nutrient Digestion, Energy Metabolism, Methane Emissions, and Rumen Microbiota in Mutton Sheep
Source: Animals (Basel). 2025 Nov 30;15(23):3460. doi: 10.3390/ani15233460 (PMC12691051; doi:10.3390/ani15233460)
Supplement: Supplementary file 1 [file animals-15-03460-s001.zip › animals-3989316-supplementary.pdf]

Supplemental table S1 Alpha diversity of ruminal bacterial communities and methanogenic archaea communities in mutton sheep fed common protein feeds

Item	Treatment[1]						SEM	*p*-value
	Control	CM	RM	DDGS	SM	FSM		
Bacteria								
Chao1	4454	1634	2016	3079	2406	3115	661.1	0.12
Observed_species	4073	1513	1819	2930	2310	3007	620.8	0.13
Shannon	9.58	7.26	7.97	8.80	8.31	8.37	0.609	0.13
Simpson	0.98	0.98	0.99	0.99	0.99	0.96	0.012	0.42
Faith_pd	252	126	147	187	171	200	35.1	0.25
Pielou_e	0.80^{a}	0.69^{b}	0.75^{ab}	0.79^{a}	0.75^{ab}	0.72^{ab}	0.030	0.01
Goods_coverage	0.98	0.99	0.99	0.99	0.99	0.99	0.003	0.08
methanogenic archaea								
Chao1	210	286	173	224	203	203	28.1	0.20
Observed_species	156	219	126	183	157	162	23.5	0.21
Shannon	4.00^{a}	3.29^{ab}	2.35^{b}	3.71^{ab}	3.33^{ab}	2.55^{ab}	0.300	0.02
Simpson	0.89	0.76	0.62	0.83	0.78	0.60	0.064	0.05
Pielou_e	0.55^{a}	0.42^{ab}	0.34^{b}	0.50^{ab}	0.46^{ab}	0.35^{b}	0.035	0.01
Goods_coverage	0.999	0.998	0.999	0.999	0.999	0.999	0.0002	0.12

[1] Control=basal diet; CM= Cottonseed meal; RM= Rapeseed meal; DDGS= Distillers dried grains with soluble; SM= Soybean meal; FSM= Fermented soybean meal. [a,b] Values in the same line with different capital letter superscripts mean samples have significant differences. SEM, standard error of the mean.

Supplemental table S2 Plasma biochemical indices in mutton sheep fed common protein feeds

Item	Treatment[1]						SEM	*p*-value
	Control	CM	RM	DDGS	SM	FSM		
TP, g/L	31.7^{d}	50.9^{ab}	45.5^{bc}	53.1^{ab}	38.4^{c}	56.9^{a}	1.84	<0.01
ALB, g/L	13.4^{c}	22.5^{ab}	21.5^{ab}	22.9^{a}	18.2^{b}	25.3^{a}	1.17	<0.01
GLB, g/L	18.4^{b}	28.4^{a}	24.0^{ab}	30.2^{a}	20.2^{b}	31.6^{a}	1.91	<0.01
TC, mmol/L	0.81^{b}	1.22^{a}	1.06^{ab}	1.30^{a}	0.83^{b}	1.25^{a}	0.134	0.04
TG, mmol/L	0.18^{b}	0.29^{ab}	0.21^{ab}	0.34^{a}	0.20^{ab}	0.24^{ab}	0.039	0.03
HDL, mmol/L	0.33^{c}	0.54^{ab}	0.54^{ab}	0.68^{a}	0.42^{bc}	0.62^{a}	0.044	<0.01
LDL, mmol/L	0.28^{bc}	0.46^{a}	0.34^{abc}	0.42^{ab}	0.28^{c}	0.37^{abc}	0.032	<0.01
vLDL, mmol/L	0.39^{b}	0.69^{a}	0.45^{ab}	0.57^{ab}	0.38^{b}	0.54^{ab}	0.081	0.03
CREA, umol/L	70.7^{c}	98.8^{a}	85.9^{ab}	87.2^{ab}	79.0^{bc}	96.0^{a}	3.33	<0.01
UREA, mmol/L	5.44^{c}	10.9^{a}	9.54^{ab}	7.50^{bc}	9.96^{a}	10.7^{a}	0.507	<0.01
GLU, mmol/L	2.86	3.56	3.15	3.60	3.11	3.99	0.291	0.11
AST, U/L	60.1^{b}	143^{a}	87.7^{b}	156^{a}	68.0^{b}	168^{a}	7.72	<0.01
ALT, U/L	5.43	8.95	8.71	8.05	9.05	7.84	2.257	0.87
ALP, U/L	47.3^{c}	95.2^{b}	96.7^{b}	96.8^{b}	73.3^{bc}	127^{a}	6.33	<0.01

[1] Control=basal diet; CM= Cottonseed meal; RM= Rapeseed meal; DDGS= Distillers dried grains with soluble; SM= Soybean meal; FSM= Fermented soybean meal. [a–d] Values in the same line with different capital letter superscripts mean samples have significant differences. SEM, standard error of the mean.

(a)

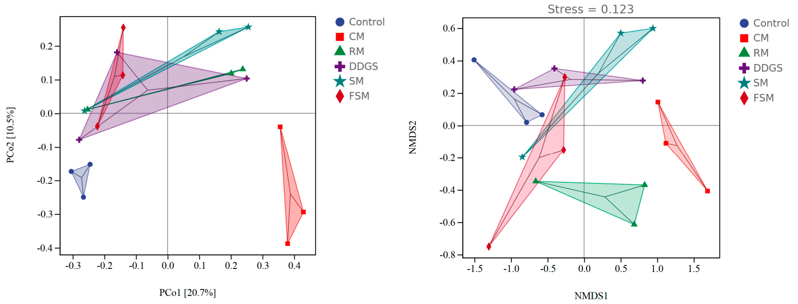

(b)

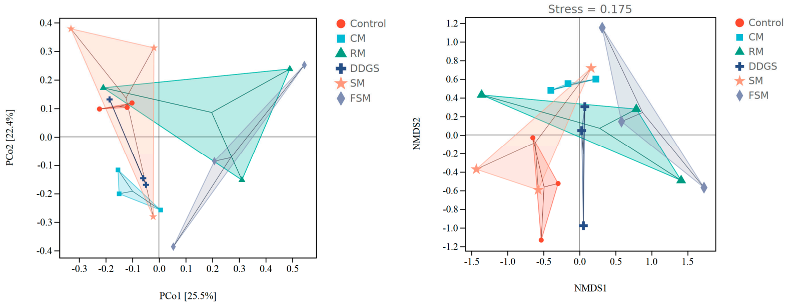

Supplemental figure S1 Beta diversity of ruminal bacterial communities (a) and ruminal methanogen communities (b) in mutton sheep fed common protein feeds
